# Alleviating sleep disturbances and modulating neuronal activity after ischemia: Evidence for the benefits of zolpidem in stroke recovery

**DOI:** 10.1111/cns.14637

**Published:** 2024-02-21

**Authors:** Zhi‐Gang Zhong, Gui‐Jin Tao, Shu‐Mei Hao, Hui Ben, Wei‐Min Qu, Feng‐Yan Sun, Zhi‐Li Huang, Mei‐Hong Qiu

**Affiliations:** ^1^ Department of Neurobiology, Institute for Basic Research on Aging and Medicine, School of Basic Medical Sciences Fudan University Shanghai China; ^2^ Department of Pharmacology, School of Basic Medical Sciences, State Key Laboratory of Medical Neurobiology and MOE Frontiers Center for Brain Science Fudan University Shanghai China

**Keywords:** c‐Fos, circadian rhythm, MCAO, sleep disorder, suprachiasmatic nucleus, zolpidem

## Abstract

**Aims:**

Sleep disorders are prevalent among stroke survivors and impede stroke recovery, yet they are still insufficiently considered in the management of stroke patients, and the mechanisms by which they occur remain unclear. There is evidence that boosting phasic GABA signaling with zolpidem during the repair phase improves stroke recovery by enhancing neural plasticity; however, as a non‐benzodiazepine hypnotic, the effects of zolpidem on post‐stroke sleep disorders remain unclear.

**Method:**

Transient ischemic stroke in male rats was induced with a 30‐minute middle cerebral artery occlusion. Zolpidem or vehicle was intraperitoneally delivered once daily from 2 to 7 days after the stroke, and the electroencephalogram and electromyogram were recorded simultaneously. At 24 h after ischemia, c‐Fos immunostaining was used to assess the effect of transient ischemic stroke and acute zolpidem treatment on neuronal activity.

**Results:**

In addition to the effects on reducing brain damage and mitigating behavioral deficits, repeated zolpidem treatment during the subacute phase of stroke quickly ameliorated circadian rhythm disruption, alleviated sleep fragmentation, and increased sleep depth in ischemic rats. Immunohistochemical staining showed that in contrast to robust activation in para‐infarct and some remote areas by 24 h after the onset of focal ischemia, the activity of the ipsilateral suprachiasmatic nucleus, the biological rhythm center, was strongly suppressed. A single dose of zolpidem significantly upregulated c‐Fos expression in the ipsilateral suprachiasmatic nucleus to levels comparable to the contralateral side.

**Conclusion:**

Stroke leads to suprachiasmatic nucleus dysfunction. Zolpidem restores suprachiasmatic nucleus activity and effectively alleviates post‐stroke sleep disturbances, indicating its potential to promote stroke recovery.

## Graphical abstract

Sleep disorders are prevalent among stroke survivors and impede stroke recovery, yet the mechanisms are unclear and no approved drugs in stroke care. We found stoke causes dysfunction of the suprachiasmatic nucleus (SCN). The hypnotic drug zolpidem restores SCN activity, and rapidly relieves post‐stroke sleep problems, implying its therapeutic promise.

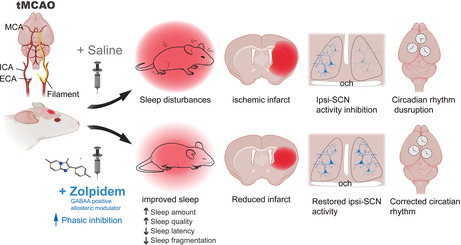

## 1 INTRODUCTION

Ischemic stroke is one of the primary diseases that endanger human health. Despite significant improvements in managing acute stroke with thrombolysis and thrombectomy, stroke remains the most common cause of long‐term adult disability worldwide.^1^, ^2^ It is of paramount relevance to promote the rehabilitation of stroke survivors, improve their quality of life and reduce the burden on their families. Though existing evidence from human studies supports at least two classes of monoaminergic drugs, serotonergic and dopaminergic, have the possibility for enhancing motor outcomes^3^, ^4^, ^5^, ^6^; however, there are currently no approved drugs to promote functional recovery after a stroke.

A large body of clinical data shows that sleep disorders are highly prevalent among stroke survivors; more than half of stroke victims have trouble sleeping in the months following. Severe abnormalities in the quantity and quality of sleep or circadian rhythm disorders occur during the hyper‐acute phase.^7^, ^8^, ^9^, ^10^, ^11^, ^12^, ^13^, ^14^, ^15^ Observational studies have shown that poor sleep quality is associated with poor functional status, which has a detrimental effect on stroke course and outcome.^16^, ^17^ Therefore, in the absence of effective means to promote stroke recovery, active interventions to improve sleep may be a promising strategy to enhance functional recovery after stroke. However, except for obstructive sleep apnea, sleep–wake disorders in stroke survivors are still often neglected in stroke patient care.^18^

Given the high prevalence of sleep disorders in stroke survivors and their negative impact on post‐stroke recovery, medications that improve sleep may benefit post‐stroke rehabilitation. Many clinical cases have shown that zolpidem, a non‐benzodiazepine hypnotic commonly prescribed to treat insomnia,^19^, ^20^ is beneficial in a large variety of neurologic disorders, though most often related to movement and consciousness disorders.^21^, ^22^ Evidence from animal studies also points to a role for zolpidem in improving behavioral recovery by modulating post‐stroke neuroplasticity,^23^, ^24^ strongly indicating the potential role of zolpidem in stroke rehabilitation. However, despite being a fast‐acting sleep‐promoting drug with few side effects, little attention has been paid to its hypnotic effect on stroke subjects. To the best of our knowledge, available data on the effects of zolpidem on sleep in patients with brain injury are from patients years after brain damage.^25^, ^26^, ^27^, ^28^, ^29^ The latest data were published by Hao et al.,^27^ in which the authors reported zolpidem‐induced EEG spectral features in eight patients (four stroke survivors) 4.5 months after brain injury. Thus, in both animal models and clinical trials, there is currently little data available on the effects of zolpidem on sleep characteristics and sleep efficiency in stroke victims during the subacute phase, a critical stage for reducing neuronal death and boosting brain repair. Furthermore, given the excitotoxic mechanism of ischemic injury, it is crucial to understand how zolpidem, a GABA signaling‐enhancing drug, affects the excitability of the ischemic neurons during the post‐stroke subacute phase.

The reason why sleep problems are so prevalent among stroke survivors is still a mystery. A study by Meng et al. in rats revealed that cortical and basal ganglia infarction resulted in immediate shifts in melatonin timing, implying that the hypothalamic suprachiasmatic nucleus (SCN), the principal circadian pacemaker in the mammalian brain, which controls the rhythmic secretion of melatonin, may be affected by transient cerebral ischemia. However, there is still a lack of direct evidence to support the notion.

Hence, the main aim of the present study was to evaluate the effects of zolpidem on sleep architecture and the EEG spectrum in rats during the subacute phase after a 30‐min middle cerebral artery occlusion (MCAO) to clarify whether zolpidem could alleviate post‐stroke sleep disturbances. Furthermore, we observed the effects of transient cerebral ischemia and zolpidem on brain activity to identify possible mechanisms by which ischemia triggers sleep disturbances and how zolpidem affects post‐stroke neuronal excitability.

## 2 MATERIALS AND METHODS

### 2.1 Animals, experimental procedures, and treatment

Male Sprague–Dawley rats (Shanghai Experimental Animal Center) weighing 250–300 g at the time of surgery were used for all experiments. Rats were individually housed under a 12:12‐h light/dark cycle (lights on at 7 a.m.), at an ambient temperature of 22°C ± 0.5°C and a relative humidity of 60% ± 2%, with ad libitum access to food and water.

**FIGURE 1:**
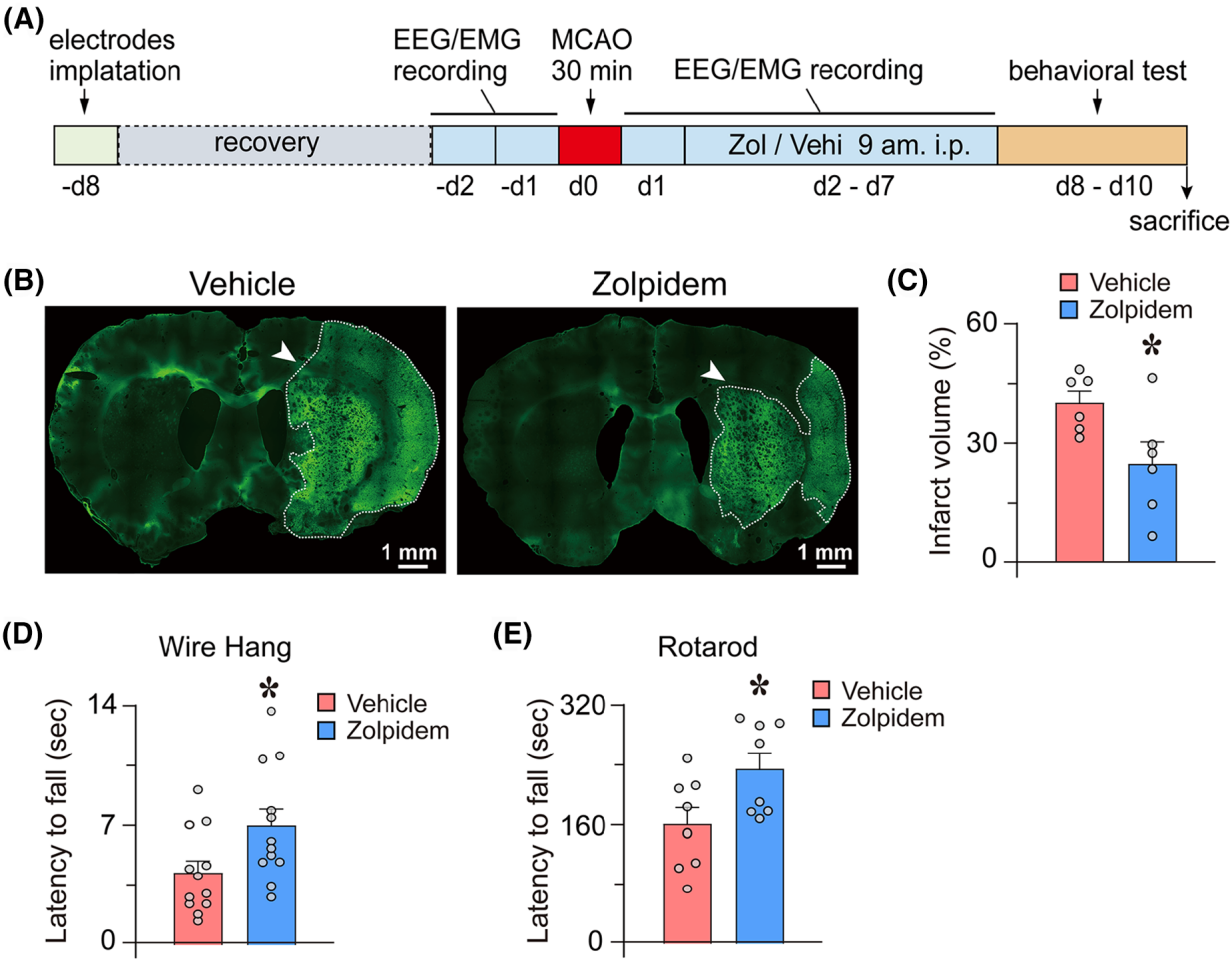
Zolpidem reduces infarct volume and improves motor functions after ischemic injury. (A) Timeline of procedures for treatments in rats. (B) Representative Fluoro‐Jade B staining in coronal sections of rats at day 10 after middle cerebral artery occlusion (MCAO). The bright green areas circled by white dotted lines indicate infarct areas. (C) Zolpidem significantly reduced the infarct volume in ischemic injured rats on day 10 after MCAO (*n* = 6). (D) and (E) The wire test (*n* = 12) and rotarod test (*n* = 8) revealed that the latencies to fall in zolpidem‐treated rats were significantly increased compared to those of the vehicle‐treated group at day 10 after MCAO. Data are presented as the mean ± SEM, *n* = 6–12/group. *p < 0.05, assessed by two‐tailed unpaired Student's *t*‐test. Vehi, vehicle; Zol, zolpidem.

All experimental protocols were approved by the Committee on the Ethics of Animal Experiments in the School of Basic Medical Science at Fudan University (Permit number: 20170223‐043). Every effort was made to minimize the number of experimental animals used and any pain and discomfort experienced by the subjects. For the continuous zolpidem administration experiment, EEG and EMG of the rats were recorded before the MCAO procedure, and EEG/EMG was continuously recorded for a further 6 days from ischemic/reperfusion day 2 (I/R d2) to I/R d7 after MCAO. Freshly prepared zolpidem or vehicle was intraperitoneally administered to the rats at 9 a.m. each day during these 6 days. Neurobehavioral tests were performed on rats during I/R d8–d10 after MCAO. Following completion of the behavioral test, the rats were euthanized, and their brain tissues were collected and assessed for brain damage (Figure 1A). In acute administration studies, zolpidem was administered at 22.5 h after the stroke. The rats in the control group were injected with the saline vehicle simultaneously, and brains were then harvested 90 min later for infarction and neuronal activity assessment.

### 2.2 EEG/EMG recordings and analysis

Rats were implanted with electrodes for EEG/EMG recordings under chloral hydrate anesthesia as previously described.^30^, ^31^ Five days after electrode implantation, each rat was transferred to the recording chamber to habituate to the recording cable and conditions for 2 days. Then, the EEG and EMG signals were continuously recorded for 48 h (serving as baseline sleep data). Each rat was then subjected to transient MCAO for 30 min, and EEG/EMG recordings were obtained from 1 to 7 days after the ischemic stroke. The recordings started at 7:00 a.m. (onset of light period). EEG/EMG signals were amplified and filtered (EEG: 0.5–30 Hz; EMG: 20–200 Hz), digitized at a sampling rate of 128 Hz, and recorded using VitalRecorder (Kissei Comtec, Nagano, Japan). When completed, EEG/EMG data were automatically scored offline in 10‐s epochs as wakefulness, non‐REM (NREM) sleep, or REM sleep in SleepSign (Kissei Comtec, Nagano, Japan). After automatic scoring, sleep–wake stages were examined and manually corrected. The durations spent in wakefulness, NREM sleep, and REM sleep were determined from the scored EEG/EMG data. EEG power spectra for each epoch were analyzed offline using fast Fourier transformation (256 points, Hanning window, 0–24.5 Hz with 0.5 Hz resolution). The circadian amplitude of wakefulness was calculated using the following formula: circadian index of wakefulness (CI_wakefulness_) = (mean_dark wakefulness_ − mean_light wakefulness_)/mean_24 h wakefulness_.^30^

### 2.3 Transient focal ischemia

Transient focal ischemia was produced by 30 min of MCAO under chloral hydrate anesthesia, as previously described.^31^ Body temperature was monitored via a rectal probe and was maintained at 37°C ± 0.5°C throughout the surgeries using a controlled heating pad, and arterial blood samples were collected to measure pCO_2_, pO_2_, and pH via an i‐STAT blood‐gas analyzer (Abbott Laboratories, Chicago, USA). Rats with normal physiological indicators were subjected to MCAO surgery. MCAO was performed mainly according to the method described by Longa et al.^32^ In brief, the left common carotid artery, external carotid artery (ECA), and internal carotid artery (ICA) were isolated. A 4‐0 nylon monofilament was introduced into the ECA lumen and was gently advanced into the ICA until slight resistance was felt. The filament was left in place for 30 min and was then withdrawn to allow reperfusion of the ischemic brain. After recovering from anesthesia, rats were returned to the recording chamber for EEG/EMG acquisition, during which they were provided food and water ad libitum.

### 2.4 Zolpidem treatment

Zolpidem (CAS NO: 99294‐93‐6, Jiaxing Aisen Chemical Co., Ltd. Jiaxing, China) was dissolved in saline and administered intraperitoneally at a dose of 10 mg/kg. In chronic administration studies, in view of sleep disturbances mainly occurring in the first week after 30‐min MCAO in our ischemic model,^31^ zolpidem was administered at 9 a.m. daily for 6 days, starting the second day after the ischemic stroke procedure (I/R d2). In acute administration studies, zolpidem was administered at 22.5 h after stroke.

### 2.5 Fluoro‐Jade B staining

Upon completing the Neurobehavioral tests, the rats were deeply anesthetized and transcardially perfused with ~50 mL saline, followed by 500 mL ice‐cold 4% phosphate‐buffered paraformaldehyde. The brains were removed, immersed in 30% sucrose at 4°C, and then sliced into four series of 40 μm coronal sections on a freezing microtome (Model 820‐II, Leica, Germany).

Fluoro‐Jade B staining was used to reveal the infarct area of each ischemic brain in chronic administration studies. In brief, brain sections from Bregma +2.0 mm to −2.0 mm, as per the atlas of Paxinos and Watson,^33^ were immersed in the following: 80% ethanol containing 1% sodium hydroxide for 5 min; 70% ethanol for 2 min; 0.06% potassium permanganate for 10 min; and Fluoro‐Jade B (4 mg/L in 0.1% acetic acid, Millipore, USA) for 20 min. The fluorescent signals were detected at an excitation wavelength of 488 nm via an Olympus VS120 slide‐scanner microscope. The infarct area of each section was measured and calculated using ImageJ software (National Institute of Health, USA). The infarction volumes are expressed as a percentage of the volume of the contralateral hemisphere.^34^

### 2.6 Wire hang test

The wire hang test was used to assess motor function, particularly muscle strength, endurance, and grip strength. On day eight after the MCAO operation, each rat was placed on a wire cage lid (30 cm width × 45 cm length) and was allowed to grasp the wire cage with all four limbs. Then, the cage was turned upside down, and each rat hung on top of the cage. The time until each rat fell from the top of the cage was recorded as the latency to falling. The procedure was repeated five times, with intervals of 15 min between each session. The average falling latency of the five trials was taken as the test data.

### 2.7 Rotarod test

The rotarod test was used to evaluate motor coordination and balance. The rats were trained on I/R d8 and I/R d9 and were tested on I/R d10. Rats were placed on a rotarod (Med Associates, Georgia, VT, USA) accelerating from 4 to 40 rpm over a 5‐min period. The procedure was repeated for three trials, each separated by a 15‐min inter‐trial interval. Each trial started at the onset of acceleration and ended when the rat fell off the rotarod. The latency to fall from the rotarod of each trial was recorded, and the test data are presented as the mean latency of the three trials.

### 2.8 Immunohistochemical staining

At 24 h after MCAO, the rats' brain slices were obtained as described in the Fluoro‐Jade B staining method. After assessing the lesion size by Cresyl violet staining, immunostaining was performed as previously described.^35^ One series of sections were processed for anti‐c‐Fos staining. The primary antibody was rabbit anti‐c‐Fos (1:5000, ab190289, Abcam), and the final step peroxidase reaction was visualized with 0.05% 3,3‐diaminobenzidine tetrahydrochloride (D5637, Sigma) in PBS and 0.01% H_2_O_2_ and strengthened with 0.002% Ni, 0.001% CoCl_2_. Sections of the striatum from another series of sections were stained with a rabbit polyclonal primary antibody against neuronal nuclei (NeuN, 1:2000, ABN78, Millipore) to value the ischemic neuronal injury, and the process was the same as the c‐Fos staining except that the peroxidase reaction was not strengthened.

For immunofluorescence labeling, brain sections of the SCN were double stained with primary rabbit anti‐c‐Fos (1:5000, ab190289, Abcam) and mouse anti‐vasoactive intestinal polypeptide (VIP, 1:500, sc‐25347, Santa Cruz). The secondary antibodies were Alexa Fluor‐488 conjugated donkey anti‐rabbit antibody (1:1000, A21206, Invitrogen) and Alexa Fluor‐594 conjugated donkey anti‐mouse antibody (1:1000, A21203, Invitrogen), respectively. The sections were then counterstained with DAPI, and fluorescence images were captured with an Olympus VS120 slide scanner microscope. The border of the SCN was determined with the aid of DAPI and VIP‐stained images.

### 2.9 Inclusion and exclusion criteria

A total of 40 rats were used in the experiment. Following the MCAO surgery and recovery from anesthesia, all rats except those with neurological scores of 0 (3 out of 37) were subjected to subsequent treatments.^32^ For the experiments with EEG/EMG monitoring, the baseline EEG of each rat was analyzed first, and those with normal sleep–wake circadian rhythms were included in subsequent experiments. One rat was excluded from the EEG spectrum analysis due to its unstable EEG drift.

### 2.10 Statistical analysis

Animals were randomly assigned into two groups and numbered individually. Data analysis was performed using GraphPad Prism 9 Software. The normality of the data was confirmed by the D'Agostino‐Pearson test, and equal variance tests were done automatically by Prism as part of the statistical analysis. All values are expressed as mean ± standard error of the mean (SEM). Statistical significance was assessed via the two‐tailed Student's *t*‐test, or one‐way ANOVA followed by Turkey's post hoc test, with *p* < 0.05 taken as the threshold of significance.

## 3 RESULTS

### 3.1 Zolpidem reduces infarct volume and promotes post‐stroke functional recovery

We first determined whether zolpidem administration for six consecutive days starting from I/R d2 affected stroke recovery. Fluoro‐Jade B staining was applied to reveal infarct volume on day 10 after transient cerebral ischemia. As shown in Figure 1B, the bright green staining indicated the infarct areas. The infarct volume of the vehicle‐treated ischemic group and the zolpidem‐treated ischemic group was 39.6% ± 2.9% and 24.2% ± 5.5%, respectively (Figure 1C). The results suggest that zolpidem treatment in the subacute phase after ischemia could significantly reduce the infarct area in rats with a 30‐min MCAO (*p* = 0.034).

Sensorimotor outcomes assessed during I/R d8–10 showed significant differences in rats' forelimb strength and motor coordination between the two treatment groups. Compared to vehicle‐treated ischemic rats, zolpidem‐treated effects exhibited a longer time in the wire hanging (zolpidem group: 6.9 ± 10 s vs. vehicle group: 4.2 ± 0.7 s, *p* = 0.029, Figure 1D) and rotarod (zolpidem group: 232.0 ± 21.3 s vs. vehicle group: 158.9 ± 21.9 s, *p* = 0.031, Figure 1E) performance. These findings indicate that neurological deficits induced by cerebral ischemia were significantly less pronounced after zolpidem treatment.

### 3.2 Zolpidem induces sleep in stroke rats and corrects post‐stroke circadian rhythm disruptions

**FIGURE 2:**
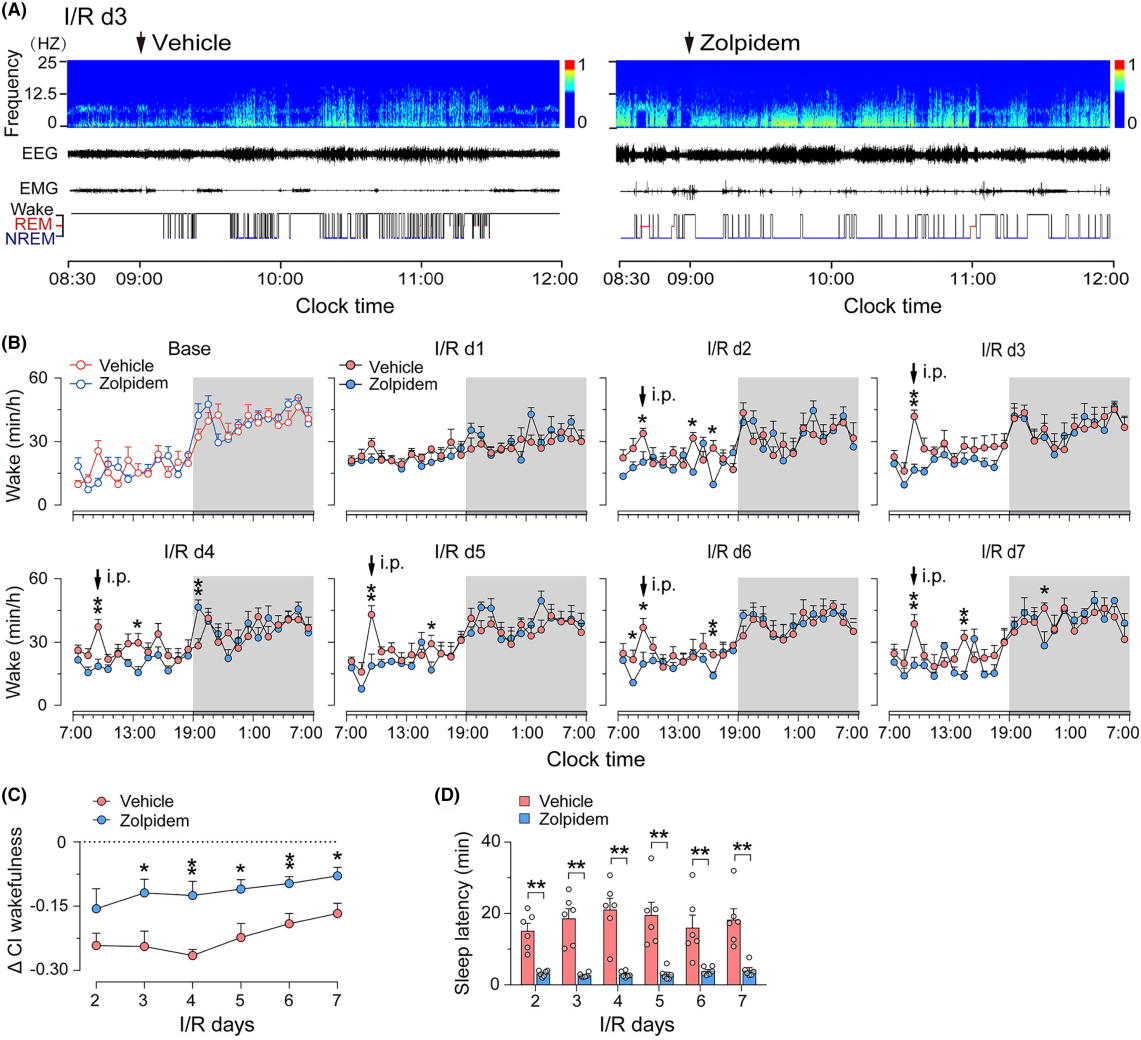
Effects of Zolpidem on the vigilance states of rats with 30‐min middle cerebral artery occlusion (MCAO) following different ischemic/reperfusion (I/R) days. (A) Examples of the relative EEG power, EEG/EMG traces, and corresponding hypnograms following vehicle or zolpidem injection at 9 a.m. in MCAO rats on I/R d3. (B) Hourly time course of wakefulness of rats in basal states, and on I/R d1–d7 after a 30 min‐MCAO. During I/R d2–d7, vehicle and zolpidem were administrated daily at 9 a.m. Each circle represents the mean hourly amount of wakefulness. The horizontal open and filled bars on the *x*‐axis indicate 12‐h light and 12‐h dark period, respectively. The gray‐shaded areas represent dark periods. (C) The changes in the CI of wakefulness (Δ CI_wakefulness_ = treatment CI_wakefulness_ − baseline CI_wakefulness_) in ischemic rats after vehicle or zolpidem treatment on I/R d2–d7. (D) non‐rapid eye movement sleep latency of ischemic rats after vehicle or zolpidem administration at 9 a.m. on I/R d2–d7. Data are presented as the mean ± SEM, *n* = 6/group. *p < 0.05, **p < 0.01, assessed by two‐tailed unpaired Student's *t*‐test between vehicle‐ and zolpidem‐treated stroke animals at each time point.

To investigate the effects of zolpidem on the sleep–wake cycles of rats suffered an acute ischemic stroke, rats were randomly assigned into two groups and daily intraperitoneally (i.p.) administered zolpidem or vehicle at 9 a.m. (inactive phase) for six consecutive days, from I/R d2 to I/R d7. The EEG/EMG of the rats were recorded simultaneously. We found that intraperitoneal injection of zolpidem at 10 mg/kg had a significant sleep‐promoting effect on ischemic rats; the rats rapidly entered NREM sleep within a few minutes and spent more time in NREM sleep than the vehicle‐treated ones. The sleep‐promoting effect of zolpidem was maintained for approximately 2–3 h (Figure 2A).

The effects of 30‐min MCAO on sleep in rats were consistent with our previous report,^31^ showing an increase and decrease in wakefulness during light and dark periods, respectively (with the most pronounced changes on I/R d1), which resulted in a flattened diurnal amplitude of the circadian rhythm, that is, a decrease of CI in stroke rats (Figure 2B,C and Figure S1A–D). As shown in Figure 2C, starting from the second dose, the reduction in diurnal amplitude in the zolpidem group was significantly smaller than that of the vehicle group (*p* < 0.05 or <0.01, unpaired *t*‐test, individual comparisons at each time point), indicating that zolpidem could relieve circadian rhythm disturbances in ischemic rats. We assessed zolpidem's impact on sleep latency as well. As shown in Figure 2D, the latency to NREM sleep in ischemic rats following zolpidem injection on I/R d2–d7 was 3.2, 2.7, 2.9, 2.9, 3.9, and 4.1 min, respectively, which were markedly shorter than the latency of 15.1, 18.6, 21.1, 19.6, 16.0 and 18.3 min in the vehicle‐treated group on corresponding I/R day (*p* < 0.01, unpaired *t*‐test, individual comparisons at each time point). Indicates that zolpidem could also accelerate the initiation of NREM sleep in rats with ischemic brain injury.

**FIGURE 3:**
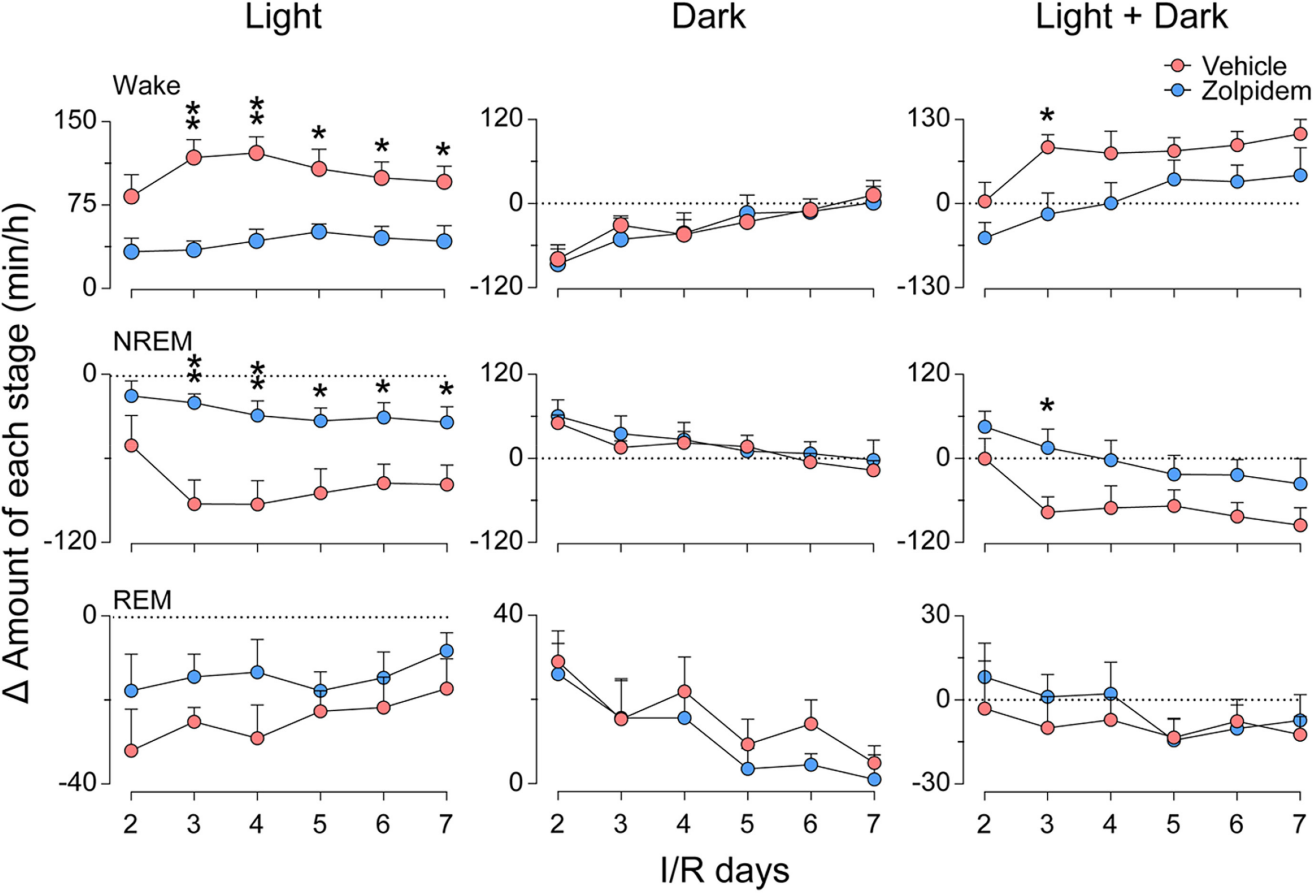
Effects of Zolpidem on changes in the amounts of each sleep/wake stage in rats with 30‐min middle cerebral artery occlusion following different I/R days. Total Δ amounts of wakefulness, REM sleep, and non‐rapid eye movement sleep during the light period, dark period, and over the 24‐h period of each I/R day, with vehicle or zolpidem daily administration at 9 a.m. Data are presented as the mean ± SEM, *n* = 6/group. *p < 0.05, **p < 0.01, assessed by two‐tailed unpaired Student's *t*‐test between vehicle‐ and zolpidem‐treated stroke animals at each time point.

We further analyzed the changes in total wakefulness, NREM sleep, and REM sleep amount during the day, night, and entire 24‐h cycle. As shown in Figure 3, during the light phase, the Δ amount of wakefulness at each I/R day in the vehicle group was far above the zero level. In contrast, during the dark phase, the Δ amount of wakefulness values was mainly negative, especially from I/R d2–d4, and returned to the baseline level on I/R d5–d7. The opposite trends were observed in the Δ amount of NREM and REM sleep (Figure 3, left and middle columns). This finding further demonstrated that 30‐min MCAO resulted in circadian rhythm disturbance in rats. The total Δ amount of each stage during the dark phase did not differ significantly between vehicle‐ and zolpidem‐treated ischemic rats (Figure 3, middle column). However, during the light period, the total Δ amount of wakefulness in zolpidem‐treated ischemic rats was markedly reduced on I/R d2 after the first dose of zolpidem and was significantly lower than that of the vehicle‐treated group from I/R d3 to I/R d7 (Figure 3, left column, upper panel). Correspondingly, the Δ amount of NREM sleep in zolpidem‐treated ischemic rats shows an opposite trend (Figure 3, left column, middle panel). The results further indicate that zolpidem quickly and effectively corrects circadian rhythm disturbance in ischemic rats.

### 3.3 Zolpidem alleviates post‐stroke sleep fragmentation

**FIGURE 4:**
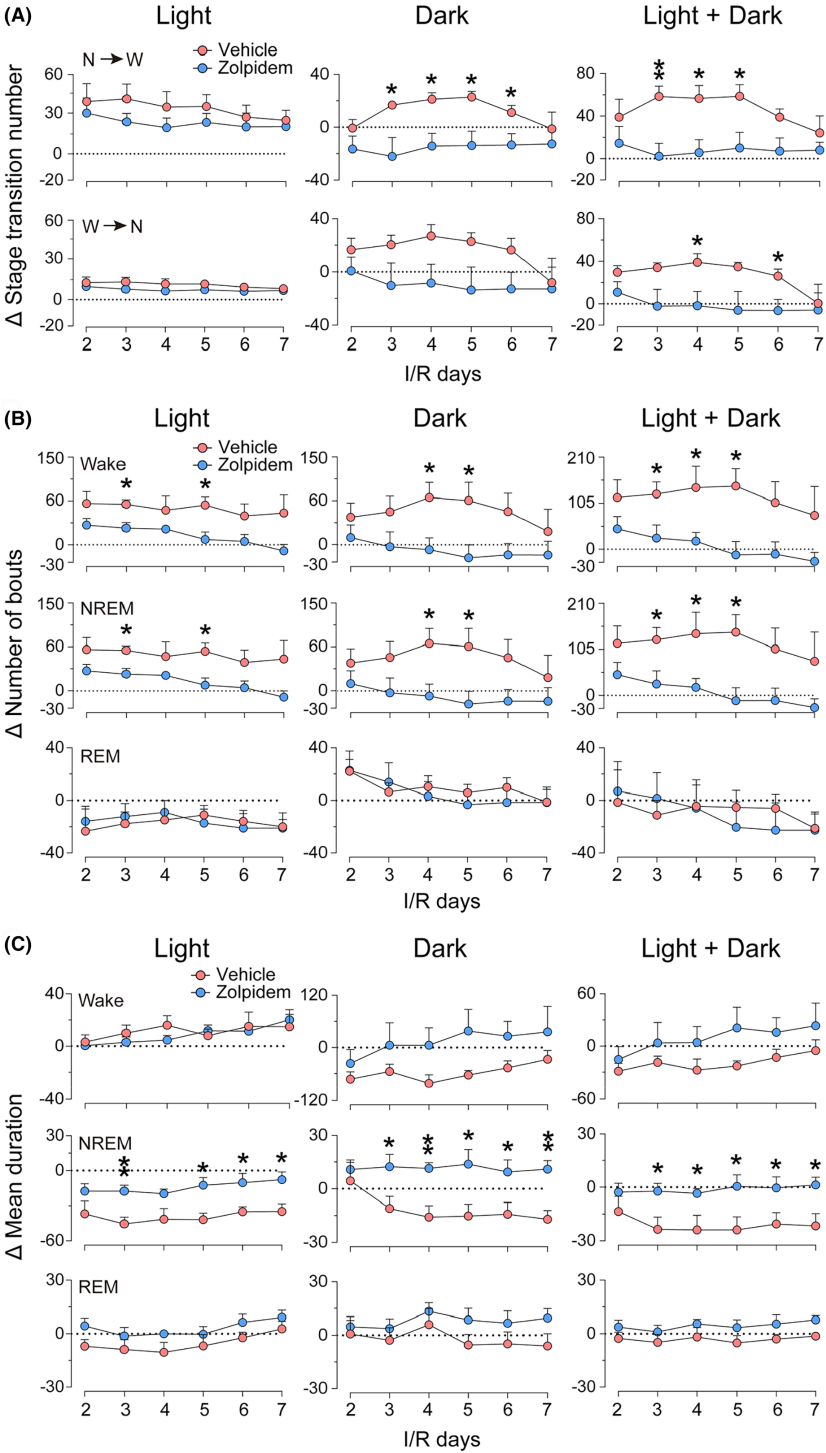
Effects of zolpidem on the characteristics of sleep–wake episodes in rats with 30‐min middle cerebral artery occlusion following different I/R days. (A) The total amount of Δ non‐rapid eye movement (NREM) sleep to wake transitions (upper panel) and Δ wake to NREM sleep transitions (lower panel) during the light period, dark period, and over the 24‐h period of each I/R day with vehicle or zolpidem daily administration at 9 a.m. (B) Total Δ number of wake, NREM sleep, and REM sleep bouts during the light, the dark period, and over the 24‐h period of each I/R day with vehicle or zolpidem daily administration at 9 a.m. (C) Δ mean duration of wake, NREM sleep, and REM sleep during the light period, dark period, and over the 24‐h period of each I/R day with vehicle or zolpidem daily administration at 9 a.m. Data are presented as the mean ± SEM, *n* = 6/group. *p < 0.05, **p < 0.01, assessed by two‐tailed unpaired Student's *t*‐test between vehicle‐ and zolpidem‐treated stroke animals at each time point.

To further understand the effects of zolpidem on sleep–wake characteristics of ischemic rats, we analyzed the stage transitions, bout numbers, and mean durations of wakefulness, NREM, and REM sleep at each experimental day and calculated the changes in these indicators of sleep (treatment value–corresponding baseline value). As shown in Figure 4A, the transitions from NREM sleep to wakefulness (N → W) in the vehicle group were significantly higher than the baseline levels in both light and dark periods from the second day after ischemia, suggesting severe sleep fragmentation in ischemic rats. There were noticeable decreases in the ΔN → W transitions in the zolpidem‐treated group during the light period, while during the dark period, both the changes in transitions from N → W and those of W → N in the zolpidem group were basically lower than the corresponding baseline level, which were completely opposite to that of the vehicle‐treated ischemic group. The changes in the numbers of wakefulness and NREM sleep bouts were roughly the same as the changes in stage transitions described above, that is, daily administration of zolpidem significantly decreased the number of stages in ischemic rats from I/R d2 to I/R d7 (Figure 4B). In contrast to the changes in stage transitions and bout numbers, the changes in the mean duration of wakefulness and NREM sleep in the vehicle‐treated ischemic rats were overall obviously lower than the baseline levels (Figure 4C). After daily administration of zolpidem, the Δ mean durations of wakefulness returned to baseline levels, and the mean duration of NREM sleep decreased much less than that of the vehicle group during the light phase, returned to the baseline level on I/R d5–d7, while during the dark period, the Δ mean durations of NREM sleep were above the baseline levels from the first dose of zolpidem.

Altogether, 30‐min MCAO resulted in severe sleep fragmentation in rats. The reductions in stage transitions and stage bouts and increases in stage durations indicated that zolpidem significantly alleviated sleep fragmentation in ischemic rats.

### 3.4 Zolpidem increases post‐stroke sleep depth

**FIGURE 5:**
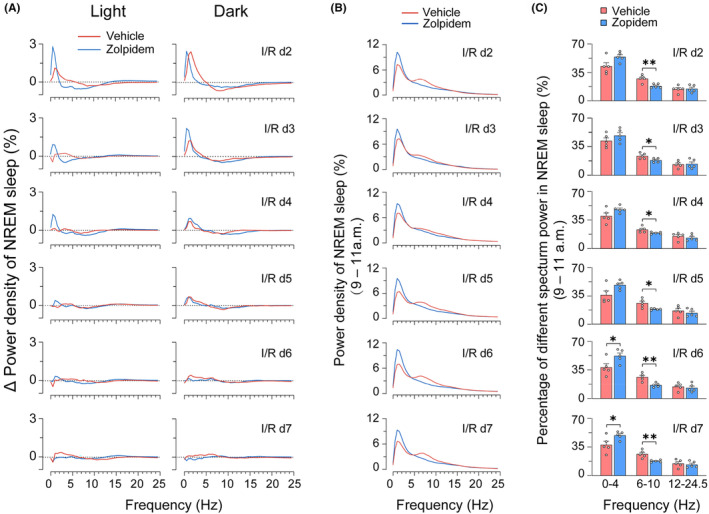
Effects of zolpidem on EEG spectral power of non‐rapid eye movement (NREM) sleep in rats with 30‐min middle cerebral artery occlusion following different I/R days. (A) Δ EEG power density of NREM sleep during light and dark periods of each I/R day, with daily administration of vehicle or zolpidem at 9 a.m. The changes in power in each frequency bin were obtained by subtracting the corresponding baseline value from the treatment value. (B) Averaged EEG power density of NREM sleep in 2 h after vehicle or zolpidem administration (9 a.m., i.p.) on each I/R day. (C) Percentage of NREM sleep power density at 0–4, 6–10, and 12–24.5 Hz within 2 h after vehicle or zolpidem administration (9 a.m., i.p.) on each I/R day. Data represented as mean ± SEM. *n* = 5/group. *p < 0.05, **p < 0.01, assessed by two‐tailed unpaired *t*‐test.

Spectral analysis of EEG power after zolpidem administration showed an increase in slow waves and a decline in theta‐alpha activity in both healthy men and patients with severe brain injury.^25^, ^26^, ^28^, ^29^, ^36^ To determine whether zolpidem also affected the EEG spectrum of ischemic rats, we evaluated changes in EEG spectral power in ischemic rats treated with the vehicle‐ or zolpidem. Data from both groups of rats showed that 30‐min MCAO significantly increased the NREM sleep power in the delta range (0–4 Hz) during the dark period of I/R d1 (Figure S2, lower panel). Consistent with our previous work, in vehicle‐treated rats, NREM sleep power in the delta range remained noticeably increased at I/R d2 and I/R d3 and returned to the baseline level by and large from I/R d4 onward (Figure 5A). Daily Zolpidem administered at 9 a.m. did not further affect the NREM sleep delta power (Figure 5A).

Given that the half‐life of zolpidem ranges from 1.5 to 3.2 h,^37^ and as shown in Figure 2A, a rapid enhancement of delta activity persisted for approximately 2 h after i.p. administration of zolpidem. Therefore, we further analyzed the NREM sleep spectrum of ischemic rats within 2 h after zolpidem or vehicle administration. From I/R d2 to I/R d7, the delta activity of NREM sleep ranged from 38.6% to 45.1% after vehicle treatment at 9 a.m. The proportion of delta activity was notably higher in the zolpidem treatment group, ranging from 51.5% to 55.6% (Figure 5B,C), indicating that zolpidem plays a role in improving sleep quality in MCAO rats. The relative theta activity (6–10 Hz), in contrast, significantly decreased within 2 h after zolpidem treatment as compared to the vehicle‐treated group (from 27.6% to 18.1%, 25.2% to 19.7%, 25.0% to 20.5%, 27.6% to 19.6%, 28.1% to 17.6%, and 28.3% to 19.0%, respectively. *p* < 0.05 or <0.01, unpaired *t*‐test). Finally, the relative alpha‐beta activity (12–24.5 Hz) did not change significantly after zolpidem treatment.

### 3.5 30‐min of MCAO inhibits the ipsilateral SCN activity

**FIGURE 6:**
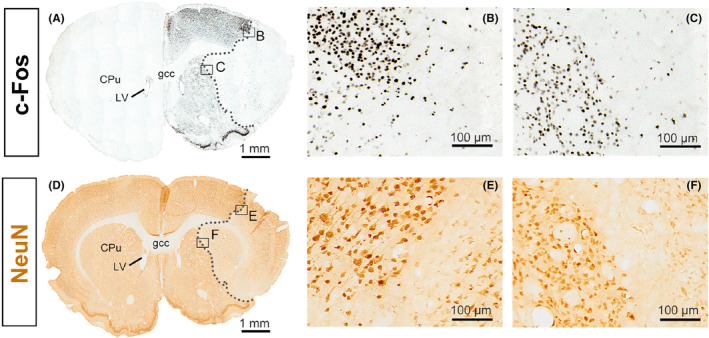
c‐Fos and NeuN immunostaining in adjacent brain slices at striatum level reveal neuronal loss in the ischemia‐involved region of rats with 30‐min middle cerebral artery occlusion (MCAO) following 24 h reperfusion. (A–C) Representative images of c‐Fos immunostaining (black) from a coronal section at the level of the striatum. The black dotted lines indicate the infarct boundary. The boxed regions in (A) are enlarged in (B) and (C), showing the cortex and striatal ischemic border zone after MCAO, respectively. (D–F) Representative images of NeuN immunostaining (brown) from an adjacent brain slice of (A), NeuN‐expression (brown color) was significantly reduced in the infarct area. The black dotted lines indicate the infarct boundary. The boxed regions in (D) are enlarged in (E) and (F), showing the cortex and striatal ischemic border zone after MCAO, respectively. Note that the infarcts elucidated by c‐Fos and NeuN immunostaining are very similar. *n* = 4. CPu, caudate putamen; gcc, genu of the corpus callosum; LV, lateral ventricle.

**FIGURE 7:**
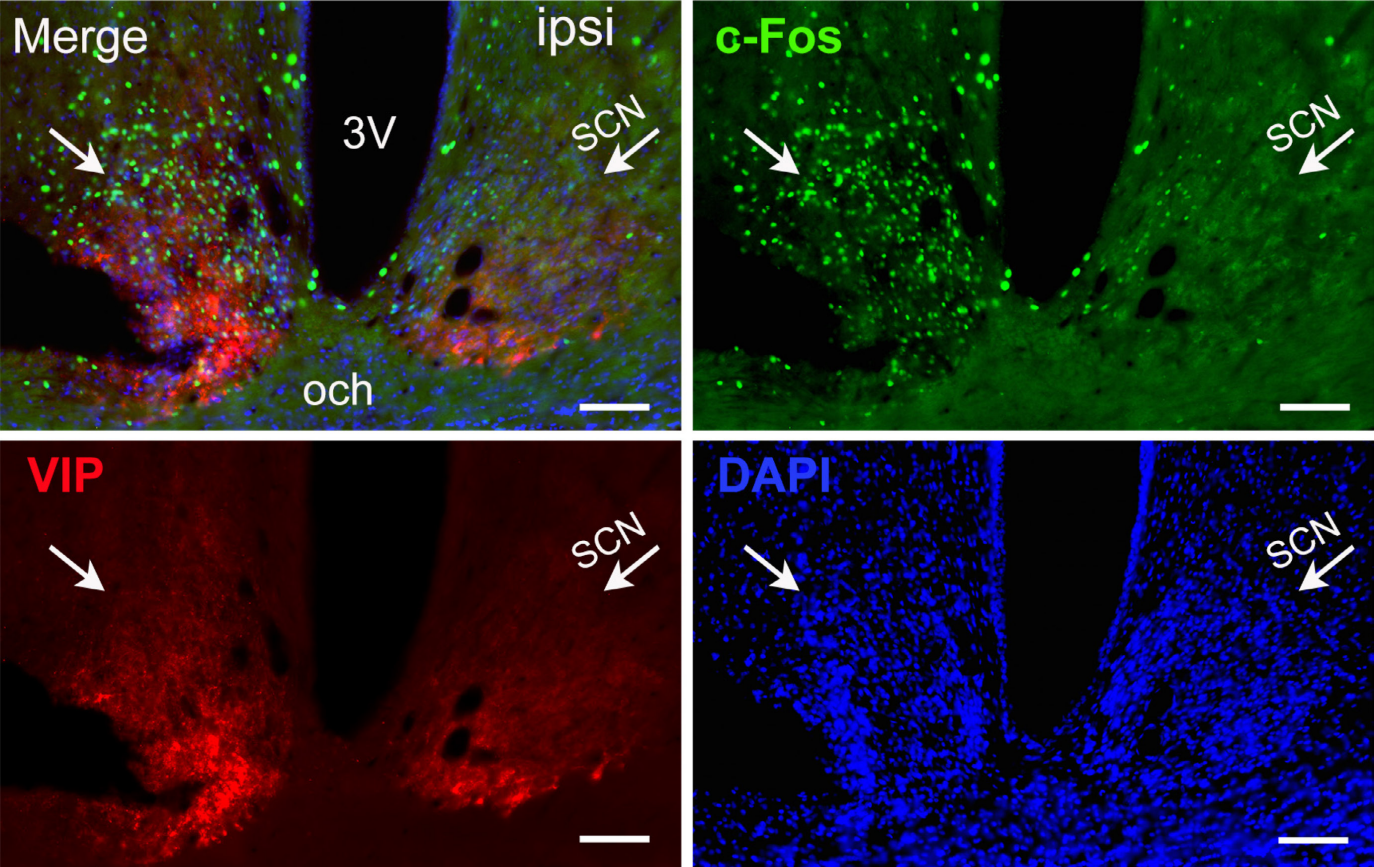
Changes of c‐Fos expression in the SCN of rats with 30‐min middle cerebral artery occlusion (MCAO) following 24 h reperfusion. Representative images of c‐Fos (green) and VIP (red) immunostaining and DAPI (blue) from a coronal section at SCN level of a rat with 30‐min MCAO following 24 h reperfusion. The border of the SCN was determined with the aid of VIP and DAPI‐stained images. Scale bars = 100 μm. *n* = 4. 3V, third ventricle; och, optic chiasm; SCN, suprachiasmatic nucleus; VIP, vasoactive intestinal polypeptide.

In our studies, the most notable alteration in MCAO rats' sleep–wake patterns is the flattened circadian rhythm, especially on the first day after ischemia. In order to uncover the underlying mechanisms, we observed the expression of immediate early gene *c‐Fos* in the whole brain of the ischemic rats on I/R d1, with particular attention to the SCN. As shown in Figure 7, C‐Fos immunoreactivity (black) was strongly upregulated in the ipsilateral cortex and striatum around the infarct area at 24 h after 30 min MCAO (Figure 6A–C), indicating strong neuronal activation in the surrounding regions of the infarct, thus contrast out the “white‐colored” ischemic area (Figure 6A). The infarcts revealed by c‐Fos immunostaining are almost identical to the ischemic neuronal loss area assessed by NeuN immunostaining (Figure 6D–F). In addition, robust c‐Fos immunoreactivity was also found in the ipsilateral thalamus (including medial and lateral geniculate nuclei), subthalamic nucleus, and substantia nigra reticulata, which are not supplied by the MCA (Figure S3). In contrast, c‐Fos immunoreactivity in the ipsilateral SCN was significantly down‐regulated (Figure 7), indicating that 30‐min MCAO leads to abnormal activity of ipsilateral SCN neurons, which results in dysfunction of the central clock located in the SCN.

### 3.6 Zolpidem restores the activity of the ischemic ipsilateral SCN

**FIGURE 8:**
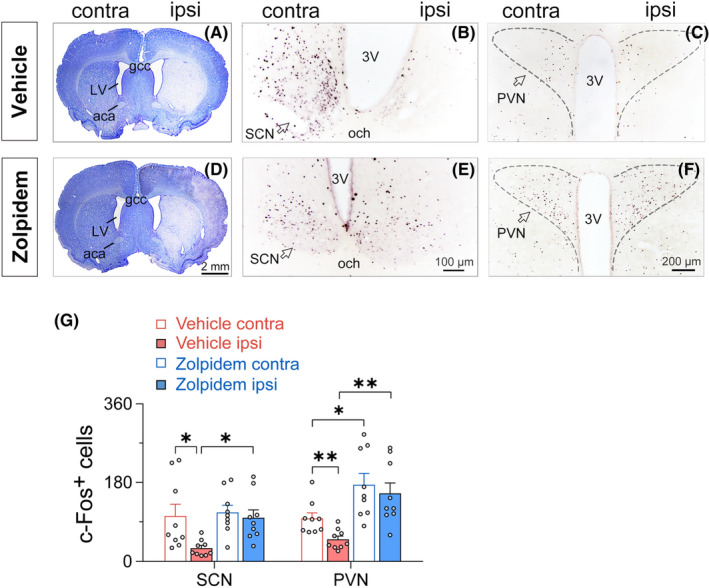
Zolpidem increases the expression of c‐Fos in the SCN and PVN in rats with 30‐min middle cerebral artery occlusion following 24 h reperfusion. Representative images of cresyl violet staining and c‐Fos immunostaining from a vehicle‐treated (A–C) or a zolpidem‐treated (D–F) rat, respectively. Vehicle or zolpidem was administrated 90 min before brain harvest at 24 h after stroke. (G) The number of c‐Fos‐immunoreactive neurons in ipsilateral/contralateral SCN and PVN after vehicle or zolpidem administration. Data are presented as the mean ± SEM, *n* = 3/group, three slices of the SCN or PVN per animal, respectively. *p < 0.05, **p < 0.01, assessed by one‐way ANOVA followed by Turkey's test (For SCN: *F*(3, 32) = 4.25, *p* = 0.0123, Turkey's Test: *p < 0.05. For PVN: *F*(3, 32) = 8.85, *p* = 0.0002, Turkey's Test: *p < 0.05 and **p < 0.01). 3V, third ventricle; aca, anterior commissure, anterior part; gcc, genu of the corpus callosum; contra, contralateral; ipsi, ipsilateral; LV, lateral ventricle; och, optic chiasm; PVN, paraventricular nucleus; SCN, suprachiasmatic nucleus.

Given that zolpidem is a GABA_A_‐positive allosteric modulator that promotes GABA signal transmission, we hypothesized that it might affect neuronal activity in the ischemic brain. CV staining showed no significant effect on ischemic infarct with a single dose of zolpidem administered within 24 h after 30‐min MCAO (Figure 8A,D). To our surprise, the c‐Fos expression in the ipsilateral SCN was dramatically increased after zolpidem treatment, with no more discernible difference from that in the contralateral side (Figure 8E,G). The results indicate that zolpidem could restore SCN activity in ischemic rats. Additionally, significantly more neurons express c‐Fos in the ipsi and contralateral paraventricular nucleus (PVN) after zolpidem administration (Figure 8F,G), which is consistent with a previous report.^38^

## 4 DISCUSSION

Sleep–wake disorders are highly prevalent among stroke survivors and negatively impact functional and structural outcomes,^16^, ^17^, ^39^ strongly implying that sleep‐improving medications should benefit post‐stroke rehabilitation. However, systematic studies that support this viewpoint are lacking. To our knowledge, this study is the first to investigate the effects of the hypnotic drug zolpidem on sleep disorders in transiently ischemic rats. We found that daily zolpidem administration during the subacute phase after a stroke quickly alleviated ischemia‐induced sleep disturbances, reduced infarct volume, and accelerated functional recovery. In addition, our study provides the first evidence of functional impairment of the SCN, the biological rhythm center located in the hypothalamus, following stroke, offering an explanation for the manifestation of disturbed sleep–wake circadian rhythms in stroke patients.

Emerging evidence reveals positive effects of GABA‐mediated inhibition on stroke outcomes in the repair phase, as demonstrated in two studies that boost phasic GABA with zolpidem, a positive allosteric GABA_A_‐α1 modulator, in the repair phase of stroke significantly enhanced functional recovery in ischemic rodents,^23^, ^24^ suggests the substantial therapeutic implications of zolpidem for stroke recovery. The two studies both focused on the influence of zolpidem on brain plasticity after ischemia and demonstrated that the beneficial effects of zolpidem on behavioral recovery were related to its promotion of neural plasticity. However, neither study addressed the impact of zolpidem on sleep in ischemic rats or mice, despite the fact that zolpidem is a commonly prescribed hypnotic drug. Our study provides the first EEG‐based evidence for the effect of zolpidem on sleep disorders in rats suffered a transient cerebral ischemia. We found that daily zolpidem administration during the sub‐acute phase of stroke not only reduced infarct volume and improved functional recovery but also rapidly alleviated ischemia‐induced sleep disturbances.

Sleep is increasingly recognized as one of the most critical factors in successful recovery following a stroke. Sleep promotion is neuroprotective during the acute phase of stroke, whereas sleep disruption after ischemia impairs functional and structural outcomes.^16^, ^17^, ^39^ We have previously shown that supplementation with melatonin, a hormone that helps control the internal clock and natural cycle of sleeping and wakefulness, reduced infarct volume and restored brain function, as well as ameliorated ischemia‐induced sleep disturbances. However, it had little effect on either the quantity or the latency of sleep.^31^ In the present study, we ascertained that the fast sleep promotor zolpidem could also rapidly induce NREM sleep in ischemic rats. After daily administration of zolpidem for six consecutive days, the circadian rhythm disturbances and sleep fragmentation in MCAO rats were significantly alleviated (Figures 2, 3, 4). Though zolpidem did not increase the power density of NREM sleep as much as melatonin did during both the light and the dark periods on I/R d2‐d5 (Figure 5), it did enhance NREM sleep delta power within 2 h after each i.p. administration (Figure 6). Strongly suggests a role for the phasic GABA signaling boosting drug zolpidem in improving sleep quality in MCAO rats. Thus, our study further supports the therapeutic implications of zolpidem in stroke management, which is associated with sleep‐related as well as plasticity‐related recovery.

The current study used immunohistochemical staining to probe changes in protein levels of immediate early gene *c‐Fos*, a neuronal activity marker, in the whole brain of rats after focal cerebral ischemic injury. Despite the difference in the temporal pattern of c‐Fos expression, the results confirm the findings of previous investigators that c‐Fos expression was increased following focal cerebral ischemic injury.^40^, ^41^, ^42^ These researchers observed c‐Fos expression after transient or permanent MCAO and found that it peaked between 1 and 4 h and returned to baseline level at 24 h after the artery occlusion. Our findings differ from these previous studies in that we show sustained, robust expression of the c‐Fos protein remained at 24 h after the onset of ischemic injury (Figure 6, Figure S3). The differences between our findings and previous studies could be attributed to differences in the severity of the ischemic injury and the post‐operative environment. A substantial body of literature supports that the entire ipsilateral hemispheric c‐Fos induction outside the infarct core is linked to cortical spreading depolarization,^43^ a phenomenon brought on by the excessive buildup of glutamic acid in the extracellular fluid after cerebral ischemia, which contributes to the expansion of ischemic infarction beyond the ischemic core.^44^ In addition, a recent study by Mehta et al. showed that the induction of c‐Fos expression after stroke could worsen ischemic brain damage by inducing the expression of many inflammatory genes. In contrast, the knockout of Fos downstream transcript could significantly lessen ischemic brain damage and facilitate neurologic recovery.^45^ These findings suggest that the strategies of inhibiting the overexcited neurons after a stroke benefit rehabilitation. Therefore, the GABA‐signaling enhancing drug zolpidem may protect ischemic penumbra in the early post‐stroke phase by counteracting glutamate‐induced excitatory toxicity.

The flattened circadian rhythm reflected by the reduction in CI of wakefulness was one of the most striking sleep–wake alterations in our MCAO ischemic model (Figure 2C and Figure S1C), which is consistent with human data on the impact of stroke on the sleep–wake cycle.^46^ What causes the abnormal circadian rhythm after a stroke is still unknown. Immediate shifts in the timing of pineal melatonin secretion in rats following MCAO,^47^ and lower melatonin concentrations in serum and urine in patients with ischemic stroke outside the retinohypothalamic pathway,^48^ suggest that, in addition to the caudate/putamen and parietal cortex, MCAO injury may also affect the hypothalamic SCN—the principal circadian pacemaker in the mammalian brain, which controls the rhythmic secretion of melatonin. However, the fact that the SCN is not supplied by the MCA leaves the assumption speculative. By in situ hybridization, Kinouchi et al.^42^ showed that immediate early genes like *c‐fos* were heavily induced in the thalamus, substantia nigra, and hippocampus of rats after MCAO, which demonstrates that widespread gene expression changes in regions far remote from the infarction and supports the above speculation on the cause of circadian rhythm disturbance after stroke. Hence, we further assess the possible changes in SCN gene expression 24 h after MCAO (the time points with the most severe circadian disturbance in our ischemic model). To our surprise, in contrast to hyperactivity in the peri‐infarct regions, the number of c‐Fos positive cells was significantly decreased in the ipsilateral SCN without significant neuronal loss following 24 h reperfusion (Figure 7). The c‐Fos protein has been demonstrated to be rhythmically expressed (higher during the day than at night) in the SCN of nocturnal rodents housed in a 12:12‐h light/dark cycle^49^, ^50^, ^51^, ^52^ (Figure S4). Thus, our present data suggest that the function of the circadian pacemaker SCN is indeed disturbed under ischemia/reperfusion conditions and is responsible for post‐stroke sleep disturbances. The actual mechanism of c‐Fos inhibition in SCN following MCAO in this study is uncertain. Since the SCN locates far from the MCA territory, one possible explanation could be part of the diffuse neurovascular injury and inflammatory responses that occur during a stroke, as evidence suggests that the immune system can modulate the circadian clock, and cytokines such as TNF‐alpha can inhibit neuronal activity in SCN.^53^ Also, given the strong induction of c‐Fos expression in peri‐infarct and remote areas in the ischemic hemisphere, it is reasonable to speculate that the decrease in the number of c‐Fos cells in ipsilateral SCN was largely attributable to the increased inhibition from its upstream. The specific upstream that may be involved needs to be further investigated.

Another surprising finding of this study was that instead of further inhibition, the GABA‐signaling‐enhancing drug zolpidem activated the ipsilateral SCN in ischemic rats (Figure 8B,E). This may be the reason why zolpidem corrects circadian rhythm disturbance in MCAO rats faster than melatonin (Figure 2C),^31^ and also suggests that zolpidem de‐inhibits SCN and restores its rhythm‐regulating function by inhibiting the SCN upstream. In addition to SCN, we also found that c‐Fos expression was significantly decreased in the ipsilateral PVN (Figure 8C,G) 24 h after the onset of MCAO; the effect of zolpidem on PVN activity was similar to but more robust than that on the SCN, as the c‐Fos expression was enormously increased in both the ipsilateral and contralateral PVN (Figure 8F,G). Recently, the neurohormone oxytocin, which is produced mainly in the PVN and acts as a neurotransmitter in the CNS, has been reported as a neuroprotective agent in ischemic injury and significantly reduces infarct in the cortex and striatum with its anti‐inflammatory and antioxidant properties,^54^, ^55^ suggest the prospective positive effects of oxytocin on the outcomes of stroke. Although we did not examine the activity of oxytocin neurons in the PVN of MCAO rats after zolpidem treatment in the present study, as in agreement with the data reported by Kiss et al.,^38^ the results of double immunostaining for c‐Fos and oxytocin in our other study showed significant activation of oxytocin neurons in the PVN by zolpidem (unpublished data). Thus, the result suggests that zolpidem can stimulate endogenous oxytocin release and raise its levels in the ischemic brain. Considering the positive effects of oxytocin on the brain, the modulation of PVN neuroactivity by zolpidem further indicates its great promise as a treatment agent for stroke rehabilitation.

## 5 CONCLUSION

In summary, our study demonstrates for the first time that enhancing phasic GABA signaling with zolpidem during the subacute phase of stroke rapidly alleviates post‐stroke sleep disturbances and speeds up functional recovery. Moreover, the results reveal for the first time that there is significant suppression of SCN neuronal activity following stroke and that such stroke‐induced suppression of the clock center can be reversed by zolpidem. Taken together, our findings shed light on the mechanisms underlying the prevalence of sleep disorders among stroke survivors, further support that GABA transmission‐enhancing drugs as a plausible strategy for improving clinical outcomes in acute stroke victims, and underscore the necessity of sleep interventions in stroke management.

## CONFLICT OF INTEREST STATEMENT

The authors report no competing interests. <Sun, Fengyan> is an Editorial Board member of CNS Neuroscience and Therapeutics and a co‐author of this article. To minimize bias, she was excluded from all editorial decision‐making related to the acceptance of this article for publication.

## Supporting information


Figure S1.–S4.

## Data Availability

The data that support the findings of this study are available from the corresponding author, upon reasonable request.
