# Supplementary material for: Alleviating sleep disturbances and modulating neuronal activity after ischemia: Evidence for the benefits of zolpidem in stroke recovery
Source: CNS Neurosci Ther. 2024 Feb 21;30(2):e14637. doi: 10.1111/cns.14637 (PMC10880125; doi:10.1111/cns.14637)
Supplement: Supplementary file 1 — Figure S1.–S4. [file CNS-30-e14637-s001.pdf]

Supplementary Material

Supplementary figures and figure legends:

Supplementary Figure 1

Data from both groups of rats showed that 30-min MCAO significantly increased and decreased the amount of wakefulness during the light and dark periods of I/R d1, respectively (**Figure S1A–B**), resulting in a significant flattening of the amplitude of sleep and wake circadian rhythms in rats, as indicated by a dramatical reduction in CI wakefulness (**Figure S1C**. CI of vehicle group: base = 0.40 ± 0.03, I/R d1 = 0.10 ± 0.03, P = 0.0002; CI of Zolpidem group: base = 0.42 ± 0.03, I/R d1 = 0.19 ± 0.03, P =0.00008). There was no significant difference in sleep-wake characteristics between the two groups in the basal state. The sleep-wake changes on the first day after ischemia were also similar in the two groups of animals.

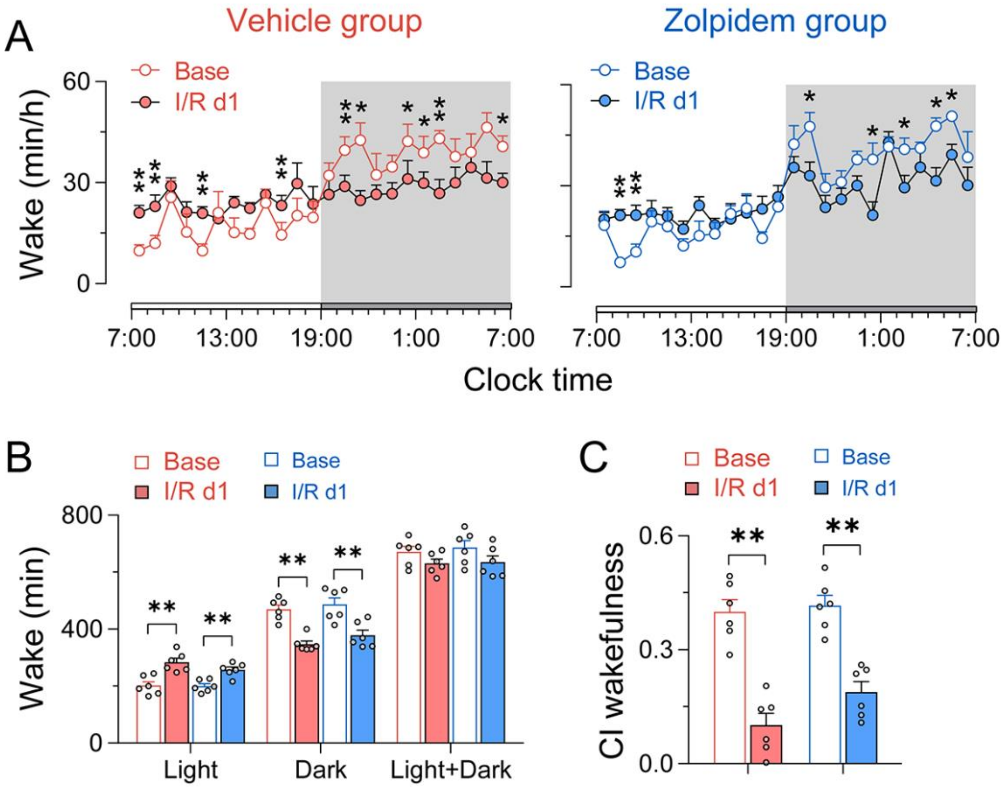

Figure S1. Changes in wakefulness and circadian rhythms in rats on I/R d1 after

30-min MCAO. (A) Hourly time courses of wakefulness across 24 h of the two groups of rats before the MCAO procedure (open circles) and on I/R d1 after 30-min MCAO (filled circles). The horizontal open and filled bars on the x-axis indicate 12-h light and 12-h dark period, respectively. The gray-shaded areas represent dark periods. **(B)** The total amount of wakefulness during the light period, dark period, and over 24-h period of rats before (open bars) and on I/R d1 after 30-min MCAO (filled bars). **(C)** Circadian index of wakefulness of rats on baseline day (open bars) and on I/R d1 after 30-minMCAO (filled bars). The data show that 30-min MCAO significantly reduced the CI of wakefulness of rats on the first reperfusion day after ischemia. Data are presented as the mean ± SE, n = 6/group. $*P < 0.05$, $**P < 0.01$, assessed by two-tailed paired *t*-test.

Supplementary Figure 2

Data from both groups of rats showed that 30-min MCAO significantly increased the NREM sleep power in the delta range (0–4 Hz) on I/R d1. There was no significant difference in the EEG spectral power of NREM sleep between the two groups of rats either in the basal state or one day after ischemia.

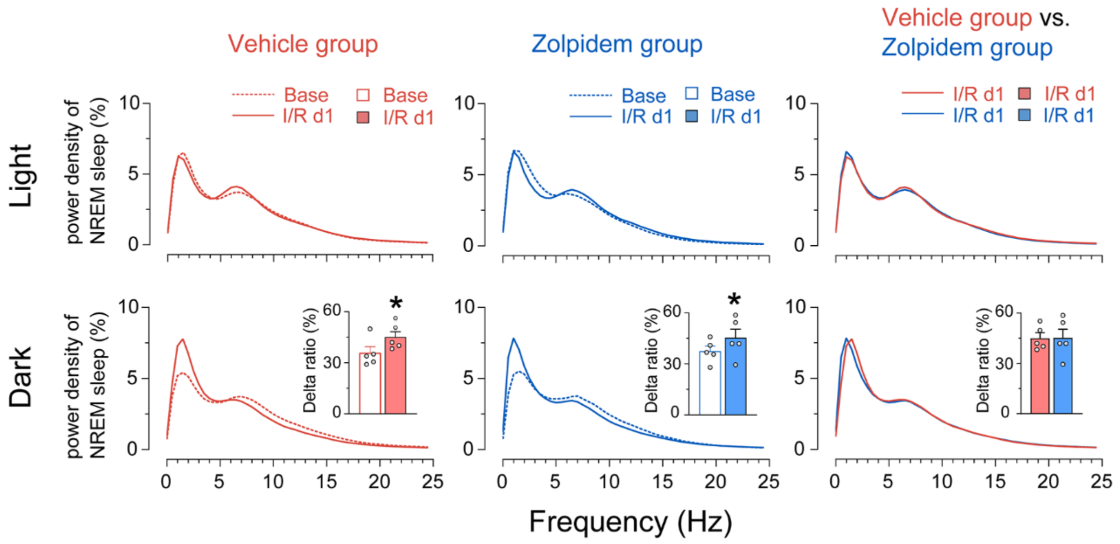

Figure S2. Changes in EEG spectral power of NREM sleep in rats on I/R d1 after

30-min MCAO. Averaged EEG power density of NREM sleep during light (upper panel) and dark period (lower panel) in rats before the MCAO procedure and one day after ischemia. The bar plots inserted in the bottom panels show the percentages of delta (0–4Hz) power of each group of rats under different conditions. Data represented as mean ± SEM. n = 5/group. * $P < 0.05$, assessed by two-tailed paired *t*-test (left and middle column) and by two-tailed unpaired *t*-test (right column).

Supplementary Figure 3

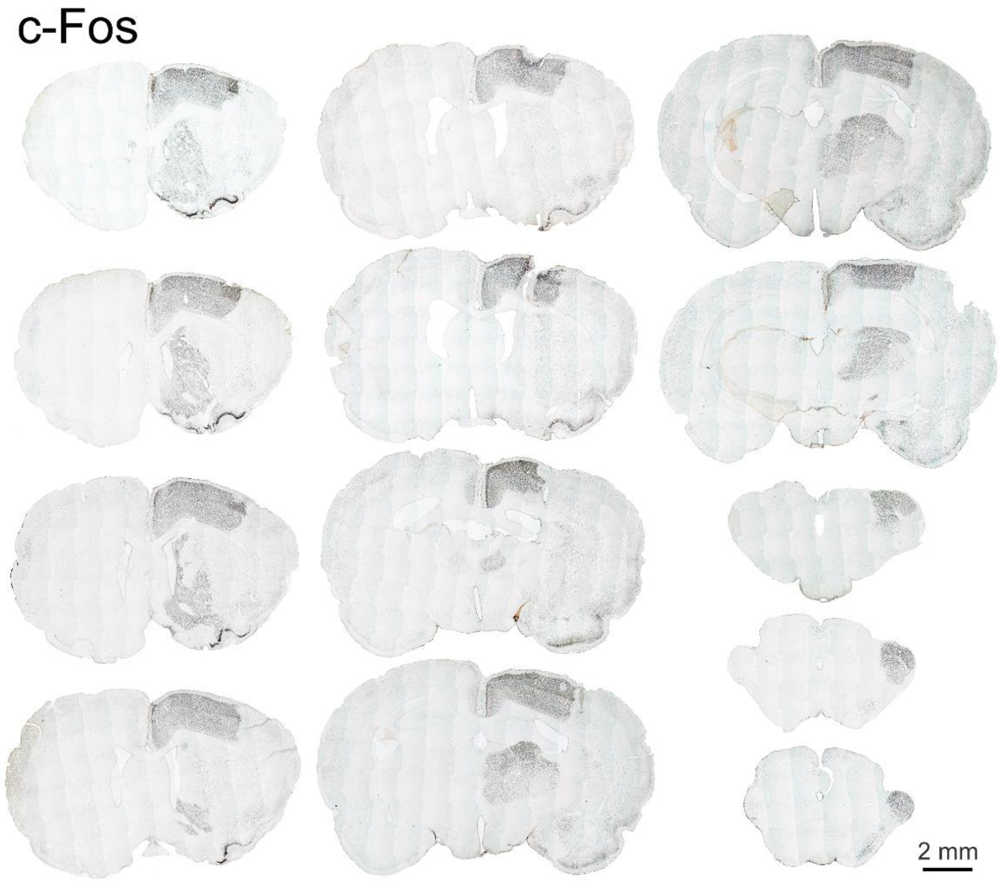

Figure S3. Changes in c-Fos expression in the brain of rats with 30-min MCAO following 24h reperfusion. Representative images of c-Fos immunostaining (black) from an ischemic rat brain. At 24 h after 30 min MCAO, c-Fos protein expression was strongly induced in the surrounding regions of the infarct in the ipsilateral cortex and

striatum. Note the induction of c-Fos protein in most of the ipsilateral thalamus including medial and lateral geniculate nuclei, subthalamic nucleus, and substantia nigra reticulata.

Supplementary Figure 4

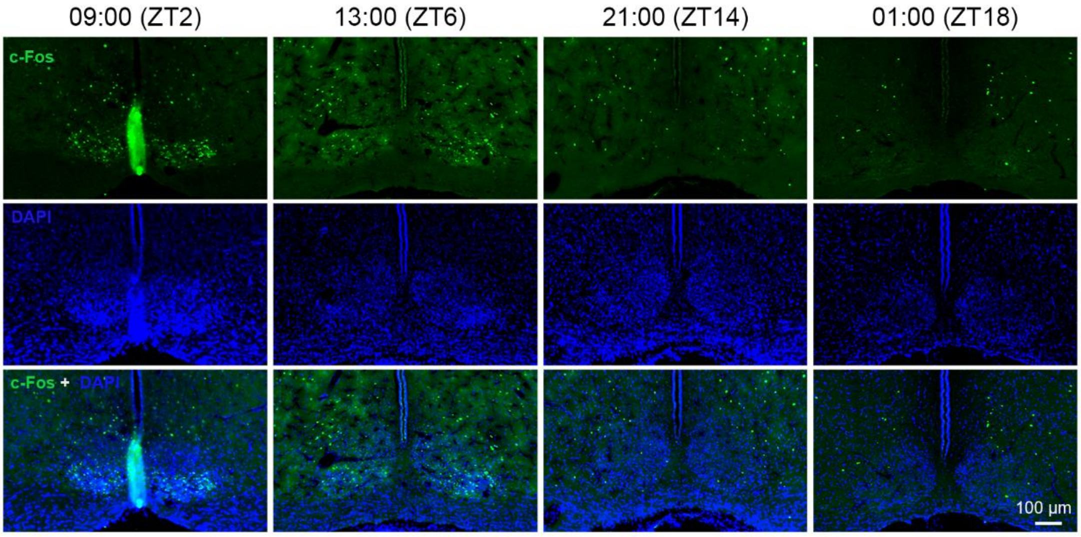

Figure S4. More SCN neurons express c-Fos during the day than during the night in rats. Representative images of c-Fos (green) immunostaining and DAPI (blue) from coronal sections at SCN level were collected at indicated times (ZT, time relative to light onset). The border of the SCN was determined with the aid of DAPI-stained images.
